# Hemoadsorption by CytoSorb in septic patients: a case series

**DOI:** 10.1186/s13054-017-1662-9

**Published:** 2017-03-27

**Authors:** Klaus Kogelmann, Dominik Jarczak, Morten Scheller, Matthias Drüner

**Affiliations:** 1 Department of Anaesthesiology and Intensive Care Medicine, Klinikum, Emden, Germany; 2 Department of Intensive Care Medicine, University Hospital Hamburg, Hamburg, Germany

**Keywords:** Inflammation, Sepsis, Septic shock, Cytokines, Cytokine storm, CytoSorb, Hemoadsorption, Hemoperfusion, Hemodynamics, Catecholamines

## Abstract

**Background:**

Septic shock, defined as life-threatening organ dysfunction caused by a dysregulated host response to infection, is a highly lethal condition that causes substantial morbidity and mortality among critically ill patients. One of the hallmarks of sepsis is the excessive release of cytokines and other inflammatory mediators causing refractory hypotension, tissue damage, metabolic acidosis and ultimately multiple organ failure. In this context, cytokine reduction by hemoadsorption represents a new concept for blood purification, developed to attenuate the overwhelming systemic levels of pro-inflammatory and anti-inflammatory mediators released in the early phase of sepsis.

**Methods:**

In the present case series, we evaluated the impact of a new hemoadsorption device (CytoSorb) used as adjunctive therapy, on hemodynamics and clinically relevant outcome parameters in 26 critically ill patients with septic shock and in need of renal replacement therapy.

**Results:**

We found that treatment of these patients with septic shock was associated with hemodynamic stabilization and a reduction in blood lactate levels. Actual mortality in the overall patient population was lower than mortality predicted by acute physiology and chronic health evaluation II (APACHE II). These effects seem to be more pronounced in patients in whom therapy started within 24 h of sepsis diagnosis, whereas a delay in the start of therapy was associated with a poor response to therapy in terms of reduction of catecholamine demand and survival. Moreover, from our patient population, medical patients seemed to benefit more than post-surgical patients in terms of survival. Treatment using the CytoSorb device was safe and well-tolerated with no device-related adverse events during or after the treatment sessions.

**Conclusion:**

Hemoadsorption using CytoSorb resulted in rapid hemodynamic stabilization and increased survival, particularly in patients in whom therapy was started early. Given the positive clinical experience of this case series, randomized controlled trials are urgently needed to define the potential benefits of this new treatment option.

## Background

Sepsis is defined as life-threatening organ dysfunction caused by a dysregulated host response to infection and its most severe state, septic shock, represents a highly lethal condition that causes substantial morbidity and mortality among critically ill patients [1]. Despite considerable advances in antibiotic therapy, resuscitative strategies, ventilator management, and improved understanding of the underlying pathophysiology, the incidence of septic shock continues to increase and remains associated with a high mortality rate, ranging from 30 to 50% and more [2, 3]. An issue that is also of concern is the escalating cost of sepsis-associated medical care, which has been calculated to reach US$17 billion annually in the USA [4] alone.

Early septic shock is predominantly the result of refractory hypotension [5] and high levels of cytokines excessively released during the exaggerated immune response immanent to the sepsis syndrome play an integral part in the collapse of the vascular system. Prolonged hemodynamic failure can cause further inflammation due to tissue damage related to ischemia/reperfusion and hypoperfusion of the vital organs. Of note, the cumulative vasopressor load in patients with sepsis is correlated with intensive care unit (ICU) mortality, the occurrence of acute circulatory failure, metabolic acidosis and renal failure [6].

In recent years, novel therapies have demonstrated a positive effect on hemodynamics and mortality [7, 8], while other approaches, despite appearing biologically rational, have less positive results.

Hemofiltration for instance, representing a widely used technique to manage renal failure, has been tested extensively in the setting of severe sepsis and septic shock [9]. Several clinical studies suggest that high-volume hemofiltration (HVHF) might decrease vasopressor requirements, and improve hemodynamics and lactate clearance [10]; however, the majority of controlled trials have not had a clinically significant and sustained effect on either cytokine removal or overall survival [11, 12].

In this context, cytokine reduction by hemoadsorption represents a new concept for blood purification, developed to attenuate the overwhelming systemic levels of pro-inflammatory and anti-inflammatory mediators released in the early phase of sepsis. Several clinical and in vitro data have demonstrated that the additional treatment with an extracorporeal cytokine adsorber results in effective removal of toxic cytokine levels and may be helpful in patients with septic multiple organ failure [13–16].

The CytoSorb whole blood adsorber is a CE-approved medical device and its use is intended in clinical situations in which cytokines are elevated. CytoSorb therapy has been safely used in more than 9000 patients. The highly porous, biocompatible polymer with its specific properties is capable of binding a broad spectrum of hydrophobic compounds with a molecular weight between 10 and 55 kDa, a range where most cytokines reside. Removal of substances is concentration dependent. While low cytokine plasma concentrations are not affected, high cytokine plasma levels are reduced effectively.

Mitzner et al. were among the first to study the effect of CytoSorb as a blood purification technique in a patient with acute-on-chronic kidney failure and septic shock [13]. To date, CytoSorb has also been successfully applied in patients with post-cardiopulmonary bypass systemic inflammatory response syndrome (SIRS) [17], rhabdomyolysis-associated myoglobinemia [18] and in liver failure [19]. Along with the effective removal of inflammatory mediators, the most prominent effect observed with the use of CytoSorb is the improvement in hemodynamics accompanied by a reduction in vasopressor doses. In the present case series, we evaluated the impact of CytoSorb, used as adjunctive therapy, on hemodynamics and clinically relevant outcome parameters in 26 critically ill patients with septic shock and in need of renal replacement therapy.

## Methods

This case series was conducted at the surgical-medical intensive care unit of Emden hospital, Germany. All patients or their relatives gave signed informed consent for retrospective data evaluation. The study protocol was approved by the Ethics Committee of the General Medical Council of Lower Saxony. We included patients with an acute physiology and chronic health evaluation II (APACHE II) score >25 and a diagnosis of septic shock.

Patients with septic shock were identified using the inclusion criteria described in Bernard et al. [20]. Briefly, patients were eligible for inclusion if they had a known or suspected infection on the basis of clinical data at the time of screening and if they met the following criteria within a 24 h period: three or more signs of systemic inflammation and the sepsis-induced dysfunction of at least two organs or organ systems. Patients excluded from analysis were pregnant or breast-feeding women, patients age <17 years and patients who were not expected to survive beyond 28 days because of an uncorrectable medical condition such as a poorly controlled neoplasm or other moribund state end-stage diseases in which death was perceived to be imminent.

Patients were initially treated following the Surviving Sepsis Guidelines. Organ failure had to include acute kidney injury (AKI) necessitating renal replacement therapy. These criteria had to be fulfilled despite maximum standard therapy including adequate fluid resuscitation (following Kidney Disease Improving Global Outcomes (KDIGO) guidelines) [21], differentiated catecholamine therapy including administration of norepinephrine to achieve mean arterial pressure (MAP) >60 mmHg, antibiotics at least 1 h after detection of septic shock (for antibiotics administered see Table 1), and lung-protective ventilation. If there was no decrease in norepinephrine demand even after an additional corticoid treatment and if the patient met the minimum criteria for AKI stage II at this stage (serum creatinine 2.0–2.9 times baseline, urine output <0.5 ml/kg/h for ≥12 h), continuous renal replacement therapy (CRRT) in combination with CytoSorb therapy was initiated.

**Table 1:** Patient characteristics, treatment modalities, clinical parameters and patient outcome

Case number	Sex	Age	Source	APACHE II	Abx	CytoSorb treatments (*n*)	Delay (h)	Cat-free days	Details on renal outcome/recovery on ICU	CRRT (days)	Ventilation (days)	Hospital stay (days)	ICU stay (days)	Predicted mortality	28-Day mortality	ICU mortality	Hospital mortality
1	M	76	Abd	45	Mero-Line-Cas	5	24	0	Non-recovery	15	17	17	17	97.5	Yes	Yes	Yes
2	M	58	Abd	48	Pip/T	3	24	7	Non-recovery	16	51	51	50	98.4	No	Yes	Yes
3	F	35	Abd	27	Pip/T-Clinda	2	24	28	Recovery	3	36	43	41	73.7	No	No	No
4	M	41	Abd	39	Mero-Fosfo	3	24	25	Recovery	13	27	36	36	94.2	No	No	No
5	F	58	Abd	27	Cefta-Levo	2	24	1	Non-recovery	2	3	4	4	73.7	Yes	Yes	Yes
6	M	75	Abd	37	Mero-Cas	3	24	0	Non-recovery	4	4	11	4	92.3	Yes	Yes	Yes
7	M	65	Abd	45	Mero	1	24	15	Non-recovery	3	3	18	18	97.5	Yes	Yes	Yes
8	M	54	Abd	37	Mero	4	24	1	Recovery	6	19	20	20	92.3	Yes	Yes	Yes
9	M	56	Abd	29	Pip/T-Clinda	3	36	3	Non-recovery	5	16	15	15	78.9	Yes	Yes	Yes
10	F	51	Abd	32	Levo-Cefta-Cas	1	48	0	Non-recovery	1	10	10	10	85.3	Yes	Yes	Yes
11	F	49	Abd	29	Mero-Line-Cas	3	48	18	Recovery	3	27	138	33	78.9	No	No	Yes
12	M	63	Abd	48	Cefta-Line-Cas	1	96	6	Non-recovery	3	14	14	14	98.4	Yes	Yes	Yes
13	M	72	Abd	34	Pip/T	2	72	4	Non-recovery	3	4	14	5	88.6	Yes	Yes	Yes
14	M	74	Pneu	33	Mero	5	24	0	Recovery	14	28	40	40	87	No	Yes	Yes
15	M	65	Pneu	33	Pip/T	3	24	0	Non-recovery	3	4	6	6	87	Yes	Yes	Yes
16	M	64	Pneu	56	Pip/T	3	24	31	Recovery	11	37	40	40	99.5	No	No	No
17	M	17	Pneu	29	Cefta-Clon	2	24	0	Unknown	2	2	2	2	78.9	No	No	No
18	F	72	Pneu	40	Pip/T-Ery	1	24	0	Non-recovery	1	1	1	1	94.9	Yes	Yes	Yes
19	M	58	Pneu	27	Pip/T-Ery	2	48	13	Chronic	9	16	43	21	73.7	Yes	Yes	Yes
20	F	79	Pneu	27	Pip/T-Ery-Cas	3	48	30	Recovery	20	34	46	40	73.7	No	No	No
21	M	62	Pneu	33	Mero-Line	3	48	2	Non-recovery	14	18	20	20	87	Yes	Yes	Yes
22	M	62	Pneu	54	Pip/T-Clinda	3	48	-	Recovery	19	33	35	35	99.3	No	Yes	Yes
23	F	53	Pneu	33	Pip/t-Ery	2	36	1	Non-recovery	3	3	4	4	87	Yes	Yes	Yes
24	M	64	Pneu	36	Mero	3	120	47	Recovery	16	45	88	72	91.2	No	No	Yes
25	M	43	Pneu	52	Pip/T-Ery-Cas	2	120	0	Chronic	3	12	12	12	99.1	Yes	Yes	Yes
26	M	46	Pneu	44	Pip/T	3	50	0	Non-recovery	7	7	7	7	97.1	Yes	Yes	Yes

*M* male, *F* female, *Cat-free* catecholamine-free, *CRRT* continuous renal replacement therapy, *Abd* abdominal focus, *Pneu* pneumonia, *Cefta* Ceftazidim, *Mero* Meropenem, *Pip/T* Piperacillin/Taz, *Clinda* Clindamycin, *Ery* Erythromycin, *Fosfo* Fosfomycin, *Cas* Caspofungin, *Line* Linezolid, *Levo* Levofloxacin, *Tyga* Tigecyclid

CRRT was performed in continuous veno-venous hemodialysis (CVVHD) mode using a citrate-based anticoagulation protocol (Multifiltrate CiCa; Fresenius Medical Care, Bad Homburg, Germany). A CytoSorb adsorber was then installed in line into the CRRT circuit in a pre-hemofilter position (AV1000; Fresenius Medical Care). Blood flow rates were kept between 100 and 150 mL/minute while dialysis doses were in the range of 20 to 30 mL/kg/h, according to standard care.

All patients received a minimum of one CytoSorb treatment and underwent additional treatments depending on their clinical response. Adsorbers were changed every 24 h or every 12 h if there was no effect or only a marginal effect within a certain amount of time (decrease of <20% in catecholamine demand within 24 h). Treatment was continued until catecholamine demand was stopped or until shock reversal, as defined by a decline in catecholamine demand to 10% of the initial dose prior to starting treatment or total cessation of catecholamines after the last CytoSorb treatment. If there was more than one CytoSorb treatment, the subsequent treatment was started immediately after termination of the previous session. The numbers of treatments are depicted in Table 1.

To assess the therapeutic impact of the combined CRRT/CytoSorb treatment we calculated or collected simplified acute physiology II (SAPS-II) score, sequential organ failure assessment (SOFA) score, the demand for norepinephrine to achieve a certain MAP (μg/h*mmHg^-1^) and assessed blood lactate levels, before and after each CytoSorb treatment. We also evaluated catecholamine-free days (in relation to ICU days) and duration of extracorporeal organ support (mechanical ventilation, CRRT). Furthermore, to differentiate the outcome of patients in relation to delay in starting CytoSorb therapy, three slots have been defined (time delay from sepsis diagnosis to start of therapy up to 24 h, between 24 and 48 h or more than 48 h). Intensive care and hospital length of stay and ICU, 28-day and hospital survival were obtained as outcome parameters. Tests of equal distribution of the variables were carried out. All sets of data were graphically presented using GraphPad Prism 5.01 software.

## Results

From March 2014 to November 2016 we treated and followed 26 consecutive patients with septic shock and the onset of at least two organ failures within the previous 48 h due to acute infection from either abdominal (post-surgical; *n* = 13) or pneumonic (medical; *n* = 13) focus, with no improvement despite adequate standard medical treatment. Patient characteristics and details on the individual sources of infection are depicted in Table 1. On admission to the ICU, the median APACHE II score was 35. All patients received at least one CytoSorb treatment and received additional treatments (up to five) according to the intensivist’s decision and as outlined in “Methods” (Table 1). The median number of CytoSorb treatments in this population was three.

**Fig. 1:**
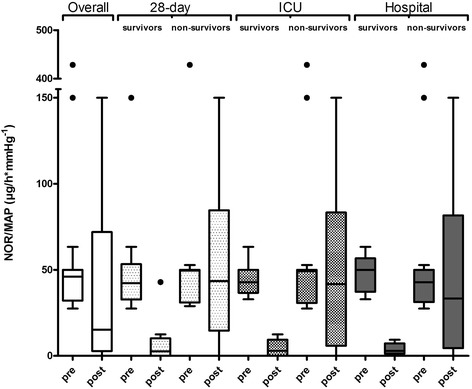
Effect of CytoSorb hemoadsorption on hemodynamics in relation to survival. Demand for norepinephrine to achieve a certain mean arterial pressure (*NOR/MAP*) (μg/h*mmHg^-1^) before (*pre*) and after (*post*) CytoSorb treatments in the overall patient population and in 28-day, ICU, and hospital survivors. In each *Tukey boxplot* the *whiskers* have equal lengths of 1.5 IQR. *Dots* represent outliers

**Fig. 2:**
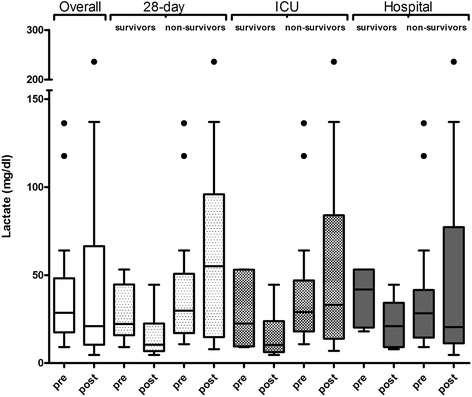
Effect of CytoSorb hemoadsorption on blood lactate levels in relation to survival. Lactate levels (mg/dl) before (*pre*) and after (*post*) CytoSorb treatments in the overall patient population and in 28-day, ICU, and hospital survivors. In each *Tukey boxplot* the *whiskers* have equal lengths of 1.5 IQR. *Dots* represent outliers

The combined application of CVVHD and CytoSorb was associated with a pronounced decrease in catecholamine demand and stabilization of hemodynamics in the overall patient population (Fig. 1, Table 2). To explain these effects, we calculated the demand for norepinephrine necessary to achieve a certain MAP by simply dividing the norepinephrine dose (μg/h) by the MAP (mmHg) measured at the same time point. In general, vasopressor dosage was clearly reduced during the treatment by 67% (Fig. 1). In parallel to the hemodynamic stabilization, the blood lactate levels decreased by 26.4% when comparing median levels before and after treatment (Fig. 2). There was a sustained reduction in the demand for norepinephrine and in lactate blood levels, even beyond 72 h after the last CytoSorb treatment.

**Table 2:** Laboratory values, catecholamine demand and scoring before and after CytoSorb treatment

Case number	CRP pre (mg/L)	CRP post (mg/L)	PCT pre (ng/mL)	PCT post (ng/mL)	Leucocytes pre (*10^3^/μL)	Leucocytes post (*10^3^/μL)	Platelets pre (*10^3^/μL)	Platelets post (*10^3^/μL)	Nor/MAP pre (μg/h*mmHg^-1^)	Nor/MAP post (μg/h*mmHg^-1^)	Lactate pre (mg/dL)	Lactate post (mg/dL)	SAPS pre	SAPS post	SOFA pre	SOFA post
1	278.9	206.1	50.9	3.53	19.09	22.96	266	238	52.8	33.3	28.3	128.6	67	60	16	13
2	196.4	318.1	4.03	2.75	20.2	15.02	342	198	32.25	1.25	34.3	10.5	42	25	10	10
3	80.9	29.01	17.5	6.25	24.75	17.88	662	53	50	0	53.1	21.1	56	35	8	10
4	135.3	296.3	49.7	18.8	10.4	15.1	211	163	63.33	2.85	41.9	23.9	51	39	11	10
5	279.3	222.2	6.95	2.18	9.44	11.02	225	165	50	45.33	47	80	59	51	11	11
6	130	60.7	-	-	11.18	36.86	362	53	28.88	83.33	29	96	51	51	8	13
7	293.6	222.5	13.6	-	6.57	12.28	408	345	30.77	69.23	20	69	59	48	15	15
8	18.4	22.7	3.28	14.8	9.79	10.59	255	132	50	26.67	31.2	8	57	40	12	10
9	24.9	91.9	36.1	5.1	10.88	11.38	370	199	30.6	0	36.1	14.8	44	42	8	5
10	133.5	109	-	-	10.94	17.09	76	59	31.7	100	136.3		53	73	17	18
11	432.7	160.2	15.2	10.1	17.04	11.27	518	215	42.85	12.5	9.2	4.7	36	33	9	7
12	209.5	50	-	33	21.04	45.61	189	7	428.5	5.88	117.7	55.1	63	38	14	14
13	241.7	214	8	1	35.9	31.3	383	319	50	16.25	13	236	62	64	13	15
14	68.1	7.6	0.2	0.9	53.18	63.16	797	758	150	42.9	21.9	22	71	62	11	11
15	51	176	2.5	11.6	12.51	9.43	157	184	50	120	10.8	137	49	48	14	13
16	129.2	95.8	0.37	0.23	17.94	8.47	266	83	41.66	2.5	18	8	57	39	12	12
17	43.4	134.5	100	229	3.95	5.2	87	77	50	5	53.2	44.6	41	22	11	9
18	27.9	35.8	-	-	14.7	15.4	163	153	49.23	150	64	44	60	-	12	12
19	231.7	260.8	12.8	0.75	9.8	15.3	62	47	50	3.07	16.1	13.5	41	43	15	10
20	339	197.8	0.9	7.7	16.37	33.7	286	186	32.85	9.41	22.6	10.4	52	55	10	7
21	114.1	302.8	1.5	43.8	27.3	32.2	271	193	36.4	41.7	12.6	15.3	49	59	14	13
22	103	44.4	101	33.5	9.4	6.9	7	34	27.5	0	18	7	60	38	20	9
23	45.9	-	3.22	1.04	5.52	6.9	108	31	30	85	52	64	48	50	16	16
24	153.3	88.3	1.37	1.87	12.6	11.04	231	202	36.66	0	9.5	6.3	58	31	10	10
25	77.7	111.2	13.8	2.1	22.07	23.64	51	38	50	80	30.6	19.1	46	46	15	15
26	290.3	426.4	21.9	15.2	13.27	22.46	79	88	41.43	14.12	26	14	38	40	16	16

*CRP* C-reactive protein, *PCT* procalcitonin, *pre* pre-treatment, *post* post-treatment, *Nor* norepinephrine, *MAP* mean arterial pressure, *SAPS* simplified acute physiology score, *SOFA* sequential organ failure assessment

We only saw marginal differences in the SAPS II (decrease of 18.1%) and SOFA scores (decrease of 4.1%) during and after therapy.

Shock reversal was observed in 10 patients (38.5%). During the course of CytoSorb treatment, all patients who survived to day 28 (*n* = 10) had reduction in catecholamine demand to 0–29.2% (median 5.3%) of their initial dose before the start of treatment, while patients who did not survive to 28 days (*n* = 16) had no reduction in the median catecholamine demand (102.6% of their initial dose). The same was true for patients surviving beyond ICU discharge (*n* = 7) (Fig. 1). Hospital survival was greater in patients who initially had higher catecholamine demand compared to their non-surviving counterparts, but in whom catecholamine dosages were reduced significantly during their treatments with continuous veno-venous hemodialysis (CVVHD) and CytoSorb. These effects were not related to a specific number of treatments (i.e. patients who had five treatments also had reversal of shock and survived).

The percentage of catecholamine-free days in the seven ICU survivors was 68.29%, whereas non-survivors only spent 7.5% of their ICU stay catecholamine-free. Of note, one patient (age 17 years) who was admitted with pneumonia and an APACHE II score of 29, and required two CytoSorb treatments to reduce his catecholamine demand to 10% of the initial dose (before starting therapy), required catecholamine support until discharge. Due to transfer to a tertiary center, he only had an ICU and hospital stay of 2 days.

ICU and hospital survivors had a lower median APACHE II score on inclusion in the study when compared to non-survivors (29 vs. 37 and 29 vs. 36, respectively). Mortality as predicted by APACHE II score in the overall patient population was 89.9%. The actual 28-day, ICU and hospital mortality was 61.54%, 73.08% and 80.77%, respectively.

**Fig. 3:**
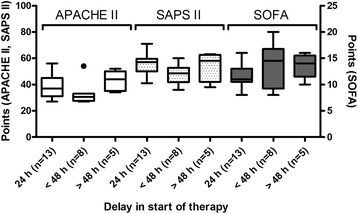
Relationship between delay in starting therapy and the severity of illness using the acute physiology and chronic health evaluation II (*APACHE II*), simplified acute physiology score II (*SAPS II*) and sequential organ failure assessment (*SOFA*) score before (*pre*) treatment. In each *Tukey boxplot* the *whiskers* have equal lengths of 1.5 IQR. *Dots* represent outliers

Groups of patients starting therapy within <24 h, between 24 and 48 h or after >48 h had a median delay of 24 h, 48 h and 96 h, respectively. Disease severity in our patients was not related to differences in delay in starting therapy, i.e. patients treated early had a median APACHE II score of 37, whereas patients starting treatment between 24 and 48 h had an APACHE II score of 30.5 and those treated after 48 h had an APACHE II score of 44 (Fig. 3). The SOFA score was lowest in the early treatment group (median SOFA score of 11) compared to the other groups (median score 14.5 in patients treated between 24 and 48 h and 14.0 in patients treated after >48 h).

The hospital mortality rate was 69.23% when the start of therapy was delayed by <24 h (92.3% predicted mortality in these patients) (Table 3). Short-term 28-day mortality in this subset of patients treated early was even lower at 53.3%. Poor outcome was more frequent in patients starting CytoSorb therapy late (87.5% of non-survivors with a delay between 24 and 48 h and 100% in non-survivors with a delay >48 h). The median start of CytoSorb therapy in hospital non-survivors was much later than in survivors (36 h vs. 24 h, respectively). All ICU survivors who had been treated early left the hospital alive, whereas none of the patients treated after a delay >48 h did. In patients in whom treatment was started early (<24 h), eventual 28-day survivors had a reduction in catecholamine demand during the course of their CytoSorb treatment(s) to 5.3% of their initial dose before starting treatment. However, non-survivors had a drastic increase in catecholamine demand at the same time (225% of their initial dose).

**Table 3:** Association between delay in start of therapy and mortality (i.e. predicted mortality, 28-day, ICU, and hospital mortality) in the overall patient population and in post-surgical and medical patients

		Predicted mortality	28-Day mortality	ICU mortality	Hospital mortality
Delay in starting therapy	<24 h (*n* = 13)	92.3	53.8	69.2	69.2
	<48 h (*n* = 8)	82.1	62.5	75.0	87.5
	>48 h (*n* = 5)	97.1	80.0	80.0	100.0
Focus	Abdominal/post-surgical	92.3	69.2	76.9	84.6
	Pneumonic/medical	87.0	53.8	69.2	76.9

Results are presented as median values

Hospital mortality was 75% (predicted mortality 93.25%) in post-surgical patients who started therapy early and it was 60% (predicted mortality 87%) in patients with medical pneumonia who started therapy early (Table 3). Reduction in catecholamine dosages was slower in medical patients when compared to surgical patients in the group of patients who started treatment early (14% vs. 40%, respectively). The median delay in starting therapy after diagnosis of sepsis was 24 h in the overall post-surgical group and 48 h in the medical group. We did not observe any device-related adverse events and there were no problems installing the adsorber in the pre-hemofilter position.

## Discussion

In the present case series, we treated severely ill patients with septic shock (APACHE II >25) who had two or more organ failures, including CRRT-dependent renal failure, with a combination of CVVHD plus hemoadsorption. Treatment was associated with rapid hemodynamic stabilization and a decrease in blood lactate. Mortality in the overall patient population was lower than mortality predicted by APACHE II scores. These effects seemed to be more pronounced in patients in whom therapy started within 24 h of sepsis diagnosis. Non-survivors had a greater delay in starting therapy (>48 h), were older, and had a poorer response to therapy in terms of reduction in catecholamine demand within a certain time frame. Moreover, among our patient population, medical patients seemed to benefit more than post-surgical patients in terms of survival.

Albeit this study is descriptive only, we observed reversal in shock (defined as free from catecholamines or a decrease to 10% of the maximum dose) in 38.5% of our patients, and all those who had a clear decrease in catecholamine demand during the course of their CytoSorb treatment(s) survived to day 28 and survived their stay in ICU. This is in line with as yet unpublished but publicly presented data from a study in a subset of patients with refractory septic shock at University Greifswald, Germany, in which a considerable number of patients were rescued from a life-threatening situation. Interestingly, ICU survivors (*n* = 7) spent 68.29% of their ICU stay catecholamine-free, whereas on average, non-survivors spent only 7.5% of their ICU stay free from catecholamines.

Our findings, therefore, are in line with several experimental and clinical data mostly from case reports and case series, which have suggested that CytoSorb might be an effective rescue therapy, stabilizing hemodynamics, decreasing vasopressor requirements, and improving lactate clearance in the setting of septic shock and post-cardiopulmonary bypass SIRS [22–25].

Our indication for CytoSorb therapy is comparable to the former indications in the PROWESS trial for activated, recombinant human protein C (drotegocin alfa activated). These criteria included at least two organ failures with an APACHE II score >25, and no decrease in the requirement for norepinephrine in spite of adequate therapy over 24 h. Of note, the APACHE II score in our subset of patients was relatively high (median 35) when compared to other studies such as the PROWESS [20] or MAXSEP trial [26], in which mean APACHE II scores were 24.6 and 21.6, respectively. Our set of patients with a median APACHE II score of 35 was predicted to have a mortality rate of 89.9%. Importantly, in our study the observed 28-day, ICU and hospital mortality was 61.54%, 73.08% and 80.77%, respectively.

We did not observe a clear improvement in SOFA scores. However, as SOFA is a morbidity severity score that relies in part on laboratory data, which may be delayed, and in which the organ function parameters may also be slow to reflect improvement in those organs, it did not reflect the real improvement in our patients [27]. Supporting this line of argument is the fact that patients treated early in our study had a median SOFA score of 11 at the start of treatment, whereas patients with intermediate (24–48 hours) and late (>48 hours) start of treatment had SOFA scores of 14.5 and 14, respectively, pointing towards a manifestation of organ dysfunction over time. Therefore, it might be reasonable to suggest that a longer period of observation of the SOFA scores might have been superior and should be considered in future studies. Moreover, renal retention parameters, as an integral part of the SOFA score, are influenced by dialysis.

A recent blinded, randomized, controlled pilot study examining the potential benefits of the CytoSorb adsorber in patients undergoing cardiopulmonary bypass (CPB) surgery found only minimal differences between the treated patients and the control group [28]. However, these were patients with only low or moderately increased risk, and who had no signs of uncontrolled hyperinflammation, either intraoperatively or postoperatively. Therefore, their predicted mortality was relatively low. On the contrary, patients in our study were severely ill with probability of mortality of up to 90%. It is assumed that Cytosorb treatment was beneficial in this cohort, as reflected by the lower observed compared to predicted mortality rate. This important difference illustrates that the true benefit of such a new treatment option, along with many others, depends on the choice of appropriate patients with appropriate indications. It further underlines why inappropriate patient selection may be responsible for the neutral or negative results in previous studies, and may have resulted in the removal of potentially effective therapies from the market [29].

Although the concept of early goal-directed therapy [7] remains a controversial topic [30, 31] and despite the fact that the original data were generated in a trial that originated in the emergency department, there could be a direct advantage in early protocol-based treatment (volume therapy, inotropy and transfusion). Similarly, early antibiotic treatment [32] has also been positively associated with survival. It is therefore not surprising that early application of other therapies in comparable patient groups have also shown potential advantages.

In fact, observations in our patients imply that starting therapy no later than 24 h after diagnosis of septic shock might be beneficial. Whether other patients could profit from this adjunctive treatment is uncertain. As with other therapies, it is of utmost importance to have the appropriate patient selection, timing of the start of therapy and the treatment duration in order to elucidate the true benefits of this new treatment option.

### Limitations

There are several limitations associated with this case series. First and most important, this is a purely descriptive case series. The lack of a control arm and the use of multiple interventions (i.e. CytoSorb, CVVHD, antibiotics, vasopressors etc.) to treat these patients with complex conditions makes it impossible to draw any definite conclusions from the data collected. A second significant limitation of this case series is the small number of patients and the fact that not all values were available for all time points due to the retrospective nature of this study analysis. Third, the follow-up period was relatively short (up to hospital discharge), therefore it is hard to validate whether the treatment might offer any long-term benefits. Last, we did not perform cytokine measurements, which is the main target of the adsorber, as these types of measurements are not routine in our institution.

The reasons for delay in starting therapy in our patients were numerous and included the late onset of renal failure when CRRT was not necessary earlier, or the late admission of the patient to the ICU. Furthermore, delay in making a definite diagnosis (either in the ward or in the ICU), and the fact that this therapy option was not commonly known to all of the physicians also resulted in further delay. All of these limitations need to be considered in the design of upcoming trials. Furthermore, one might argue that with our kind of protocol, to wait for acute renal failure to occur might result in patients having a high probability of failure to respond to therapy. In fact, the mortality rate in AKI as part of multiple organ failure in the setting of sepsis is 76% [33].

Another concern could be whether we treated patients who would have survived without treatment anyway, if we treated those without AKI. This objection is an important issue and can be only sufficiently addressed by randomized controlled trials. Likewise, the question as to what is the appropriate dosage and duration of therapy remains unanswered, though it seems reasonable from the data gained so far that treatment should be continued until hemodynamic stabilization is achieved.

## Conclusion

To our knowledge this is the first case series reporting the use of CytoSorb therapy in severely ill patients with septic shock of two different origins. Treatment of these patients with a combination of CytoSorb and CVVHD was associated with clear stabilization in hemodynamics and concomitant decrease in vasopressor doses. Observed mortality in the overall patient population was lower than the mortality predicted by APACHE II scores. These favorable effects seem to be more pronounced in patients in whom therapy was started early after diagnosis of sepsis, whereas a delay in starting therapy was associated with a poor response to therapy in terms of reduction in catecholamine-demand and poor survival. Therefore, starting therapy early (preferably within less than 24 h after onset of septic shock) seems to potentially have advantages in terms of survival, and non-surgical, medical patients seem to benefit more. Treatment using the CytoSorb device was safe and well-tolerated with no device-related adverse events during or after the treatment sessions. Given the positive clinical experience of this case series, randomized controlled trials are urgently needed to define the potential benefits of this new treatment option.

## Abbreviations

AKI: Acute kidney injury
APACHE II: Acute physiology and chronic health evaluation
CPB: Cardiopulmonary bypass
CRP: C-reactive protein
CRRT: Continuous renal replacement therapy
CVVHD: Continuous veno-venous hemodialysis
HVHF: High-volume hemofiltration
ICU: Intensive care unit
kDa: kilodalton
MAP: Mean arterial pressure
PCT: Procalcitonin
SAPS II: Simplified acute physiology score II
SIRS: systemic inflammatory response syndrome
SOFA: sequential organ failure assessment

## References

[1] Singer M, Deutschman CS, Seymour CW, Shankar-Hari M, Annane D, Bauer M (2016). The third international consensus definitions for sepsis and septic shock (Sepsis-3). JAMA..

[2] Angus DC, Van der Poll T (2013). Severe sepsis and septic shock. N Engl J Med..

[3] Engel C, Brunkhorst FM, Bone HG, Brunkhorst R, Gerlach H, Grond S (2007). Epidemiology of sepsis in Germany: results from a national prospective multicenter study. Intensive Care Med..

[4] Coopersmith CM, Wunsch H, Fink MP, Linde-Zwirble WT, Olsen KM, Sommers MS (2012). A comparison of critical care research funding and the financial burden of critical illness in the United States. Crit Care Med..

[5] Mackenzie I (2001). The haemodynamics of human septic shock. Anaesthesia..

[6] Dünser MW, Ruokonen E, Pettilä V, Ulmer H, Torgersen C, Schmittinger CA (2009). Association of arterial blood pressure and vasopressor load with septic shock mortality: a post hoc analysis of a multicenter trial. Crit Care..

[7] Rivers E, Nguyen B, Havstad S, Ressler J, Muzzin A, Knoblich B, Early Goal-Directed Therapy Collaborative Group, et al. Early goal-directed therapy in the treatment of severe sepsis and septic shock. N Engl J Med. 2001;345:1368–1377 3 10.1056/NEJMoa010307 11794169

[8] Dellinger RP, Carlet JM, Masur H, Gerlach H, Calandra T, Cohen J (2004). Surviving sepsis campaign guidelines for management of severe sepsis and septic shock. Intensive Care Med..

[9] Honore PM, Joannes-Boyau O (2004). High volume hemofiltration (HVHF) In sepsis: a comprehensive review of rationale, clinical applicability, potential indications and recommendations for future research. Int J Artif Organs..

[10] Cornejo R, Downey P, Castro R, Romero C, Regueira T, Vega J (2006). High-volume hemofiltration as salvage therapy in severe hyperdynamic septic shock. Intensive Care Med..

[11] Tolwani A (2012). Continuous renal-replacement therapy for acute kidney injury. N Engl J Med..

[12] Payen D, Mateo J, Cavaillon JM, Fraisse F, Floriot C, Vicaut E (2009). Impact of continuous venovenous hemofiltration on organ failure during the early phase of severe sepsis: a randomized controlled trial. Crit Care Med..

[13] Mitzner SR, Gloger M, Henschel J, Koball S (2013). Improvement of hemodynamic and inflammatory parameters by combined hemoadsorption and hemodiafiltration in septic shock: a case report. Blood Purif..

[14] Hetz H, Berger R, Recknagel P, Steltzer H. Septic shock secondary to ß-hemolytic streptococcus-induced necrotizing fasciitis treated with a novel cytokine adsorption therapy. Int J Artif Organs. 2014;37:422–6.” 10.5301/ijao.5000315 24811308

[15] Born F, Pichlmaier M, Peterss S, Khaladj N, Hagl C. Systemic inflammatory response syndrome in der Herzchirurgie: Neue Therapiemöglichkeiten durch den Einsatz eines Cytokin-Adsorbers während EKZ. Kardiotechnik 2014;41–6. German.

[16] Kellum JA, Song M, Venkataraman R (2004). Hemoadsorption removes tumor necrosis factor, interleukin-6, and interleukin-10, reduces nuclear factor-kappaB DNA binding, and improves short-term survival in lethal endotoxemia. Crit Care Med..

[17] Traeger K, Fritzler D, Fischer G, Schröder J, Skrabal C, Liebold A (2016). Treatment of post-cardiopulmonary bypass SIRS by hemoadsorption: a case series. Int J Artif Organs..

[18] Suefke S, Sayk F, Nitschke M (2016). Hemoadsorption in infection-associated rhabdomyolysis. Ther Apher Dial..

[19] Frimmel S, Schipper J, Henschel J, Yu TT, Mitzner SR, Koball S (2014). First description of SPAD combined with cytokine adsorption in fulminant liver failure and hemophagocytic syndrome due to generalized HSV-1 infection. Liver Transpl..

[20] Bernard GR, Vincent JL, Laterre PF, LaRosa SP, Dhainaut JF, Lopez-Rodriguez A (2001). Efficacy and safety of recombinant human activated protein C for severe sepsis. N Engl J Med..

[21] KDIGO AKI Work Group (2012). KDIGO clinical practice guideline for acute kidney injury. Kidney Int Suppl..

[22] Peng ZY, Carter MJ, Kellum JA (2008). Effects of hemoadsorption on cytokine removal and short-term survival in septic rats. Crit Care Med..

[23] Träger K, Fritzler D, Fischer G, Schröder J, Skrabal C, Liebold A (2016). Treatment of post-cardiopulmonary bypass SIRS by hemoadsorption: a case series. Int J Artif Organs..

[24] Bruenger F, Kizner L, Weile J, Morshuis M, Gummert JF (2015). First successful combination of ECMO with cytokine removal therapy in cardiogenic septic shock: a case report. Int J Artif Organs..

[25] Hetz H, Berger R, Recknagel P, Steltzer H (2014). Septic shock secondary to β-hemolytic streptococcus-induced necrotizing fasciitis treated with a novel cytokine adsorption therapy. Int J Artif Organs..

[26] Brunkhorst FM, Oppert M, Marx G, Bloos F, Ludewig K, Putensen C (2012). Effect of empirical treatment with moxifloxacin and meropenem vs meropenem on sepsis-related organ dysfunction in patients with severe sepsis: a randomized trial. JAMA..

[27] Vincent JL, Moreno R, Takala J, Willatts S, De Mendonça A, Bruining H (1996). The SOFA (Sepsis-related Organ Failure Assessment) score to describe organ dysfunction/failure. On behalf of the Working Group on Sepsis-Related Problems of the European Society of Intensive Care Medicine. Intensive Care Med.

[28] Bernardi MH, Rinoesl H, Dragosits K, Ristl R, Hoffelner F, Opfermann P (2016). Effect of hemoadsorption during cardiopulmonary bypass surgery - a blinded, randomized, controlled pilot study using a novel adsorbent. Crit Care..

[29] Ranieri VM, Thompson BT, Barie PS, Dhainaut JF, Douglas IS, Finfer S (2012). Drotrecogin alfa (activated) in adults with septic shock. N Engl J Med..

[30] Peake SL, Bailey M, Bellomo R, Cameron PA, Cross A, Delaney A (2009). Australasian resuscitation of sepsis evaluation (ARISE): a multi-centre, prospective, inception cohort study. Resuscitation..

[31] Yealy DM, Kellum JA, Huang DT, Barnato AE, Weissfeld LA, ProCESS Investigators (2014). A randomized trial of protocol-based care for early septic shock. N Engl J Med..

[32] Kumar A, Roberts D, Wood KE, Light B, Parrillo JE, Sharma S (2006). Duration of hypotension before initiation of effective antimicrobial therapy is the critical determinant of survival in human septic shock. Crit Care Med..

[33] Liaño F, Junco E, Pascual J, Madero R, Verde E (1998). The spectrum of acute renal failure in the intensive care unit compared with that seen in other settings. The Madrid Acute Renal Failure Study Group. Kidney Int Suppl.

